# Beyond Procedural Interculturality: Indigenous Governance in Mental Health Implementation: Comment on "Routes of Well-Being, Spiritual Harmony and Recovery in Mental Health: The Community as a Policy-Maker"

**DOI:** 10.34172/ijhpm.10071

**Published:** 2026-07-25

**Authors:** Laura Inés Plata-Casas, Andrés Camilo Delgado-Reyes

**Affiliations:** ^1^National Superintendency of Health, Bogotá, Colombia.; ^2^University of Manizales, Manizales, Colombia.

**Keywords:** Coproduction, Intercultural Mental Health, Epistemic Justice, Indigenous Knowledge, Implementation Science, Governance

## Abstract

This commentary examines Agudelo-Hernández and Giraldo-Álvarez’s article as an important contribution to intercultural mental health policy in Colombia. The study illustrates how coproduction can move beyond consultation by translating community meanings of mental health into a territorial route with political relevance and administrative uptake. However, this achievement should be understood as an initial step rather than evidence of full epistemic transformation. Using Colombia’s Indigenous Intercultural Health System (Sistema Indígena de Salud Propia e Intercultural, SISPI) as a policy lens, we argue that intercultural implementation requires Indigenous decision-making authority, protection of ancestral knowledge, sustainable financing, and community-defined indicators of recovery, harmony, and collective well-being. Yet transformative effects cannot be assumed in advance. Current documentation on SISPI provides limited empirical evidence regarding operationalization, financing mechanisms, territorial coordination, and measurable outcomes, underscoring the need for ongoing evaluation as intercultural governance structures move into practice.

 The article by Agudelo-Hernández and Giraldo-Álvarez offers a significant contribution to intercultural mental health policy by showing that communities can be more than consulted stakeholders: they can become policy-makers. This contribution can be interpreted as an advance beyond consultation-based intercultural approaches, based on the processes described in the article. The Riohacha route did not merely gather community views or validate technical proposals; it incorporated community meanings into an administratively adopted pathway with political implications, although formal adoption does not in itself guarantee epistemic redistribution. In Riohacha, Colombia, the authors describe a coproduced route for mental health, well-being, and spiritual harmony, built through participatory action research with Indigenous peoples, children and adolescents, migrants, community leaders, the education sector, health actors, and local decision-makers.^1^ Its central value lies not only in documenting participation, but in showing how community definitions can become politically consequential. The study therefore raises a broader implementation question: can health systems incorporate Indigenous epistemologies without reducing them to administratively manageable categories?

 Three concepts guide this argument, used here as operational lenses rather than exhaustive definitions. Procedural interculturality refers to the inclusion of Indigenous language, actors, symbols, or practices in formal health processes without transferring authority over decisions, resources, indicators, or evaluation criteria, drawing on Walsh’s formulation of critical interculturality.^2^ Epistemic redistribution refers to changes in who has authority to define problems, legitimate knowledge, determine what counts as evidence, and evaluate whether implementation has succeeded, following Fricker and de Sousa Santos,^3,4^ Community epistemologies refer to locally situated knowledge systems through which communities define suffering, care, territory, relationality, recovery, and collective well-being, as discussed by Smith and others.^4,5^

 The answer depends on how interculturality is understood. In the original article, mental health is not framed only as symptom reduction, access to services, or adherence to clinical care. In the article, spiritual harmony is described as a relational ontology: a balance among bodies, spirits, territory, identity, autonomy, worldview, and collective life. This framing emphasizes that suffering and recovery are understood relationally rather than solely as individual psychological states. This definition challenges biomedical ontologies of mental disorder because it does not locate suffering only inside the individual. Spiritual disharmony may refer to ruptures in relationships with family, community, sacred places, ancestors, collective norms, and territory. Care may therefore require harmonization practices, spiritual authorities, family and community restoration, territorial reconnection, and culturally situated accompaniment. This is not a cultural accessory to mental healthcare. It is an organizing concept for recovery, governance, and the legitimacy of care.

 The article also demonstrates that coproduction can operate as governance rather than as consultation. Its methodological strength is the combination of participatory action research, public health participatory mapping, community-based rehabilitation, and global mental health approaches. The result was not only qualitative evidence, but a route formally adopted through a local administrative act.^1^ This matters because participatory processes often stop at listening exercises. Communities are asked to narrate barriers, validate routes, or identify needs, while the architecture of policy remains controlled by technical and administrative actors. The Riohacha experience moves further by allowing community meanings to shape the route itself. Yet the same experience exposes the fragility of this advance: formal adoption does not guarantee epistemic redistribution.

 A central risk is administrative translation. Health systems often integrate primarily what can be coded, audited, financed, standardized, and evaluated through existing institutional categories. When Indigenous knowledge enters the system only through these filters, interculturality can become procedural rather than transformative. In such cases, spiritual harmony may be recognized as a term, but not as an ontology. Traditional practices may be included as activities, but not as knowledge systems with their own criteria of indication, authority, temporality, and effectiveness. This is why the translation of Western health benefit procedures into traditional actions for mental health is one of the article’s strongest contributions and, at the same time, one of its most delicate points.^1^ It creates a practical bridge between institutional procedures and traditional care, but that bridge can become reductive if traditional actions are legitimized only when they are converted into equivalents of Western services.

 This problem is not unique to Colombia. Global mental health has increasingly emphasized community-based care, rights-based approaches, and sustainable development,^6^ while decolonial critiques have questioned whether global health can transform its own hierarchies of knowledge and power.^7^ In implementation science, the problem is even sharper: frameworks focused on barriers, facilitators, fidelity, adaptation, scale-up, and outcomes can obscure who defines feasibility, whose evidence counts, and whose feedback changes decisions.^8,9^ Without a power lens, intercultural implementation may reproduce the same hierarchy it claims to correct. It may include Indigenous voices as data while excluding Indigenous authorities from authorship, governance, financing decisions, information systems, and evaluation.

 Comparative experiences, such as First Nations health governance in British Columbia and Māori health reforms in Aotearoa/New Zealand, indicate that intercultural implementation tends to advance only when institutional mechanisms allow Indigenous authorities to shape priorities, resources, budget allocation decisions, implementation oversight, and evaluation criteria.^10^ Aotearoa/New Zealand offers an instructive contrast. The Pae Ora (Healthy Futures) Act created Iwi-Māori Partnership Boards to represent local Māori perspectives in relation to *hauora*, but the subsequent disestablishment of the Māori Health Authority illustrates that Indigenous health governance arrangements can remain politically vulnerable when decision-making authority is not durably protected.^11^ These examples do not provide simple templates for Colombia; rather, they suggest that intercultural implementation is more likely to be effective when Indigenous authority is institutionally recognized, not merely culturally acknowledged.

 Colombia’s Indigenous Intercultural Health System (Sistema Indígena de Salud Propia e Intercultural, SISPI) therefore represents a decisive yet still early-stage test for the country. Decree-Law 480 of 2025 establishes SISPI as a State-level health policy for Indigenous peoples, grounded in autonomy, self-government, ancestral knowledge, territoriality, traditional authority, and coordination with national institutions.^12^ Its legal recognition marks an important institutional shift, but its practical effects will depend on how these principles are operationalized, financed, and protected within implementation processes. This is more than a culturally differentiated component within the existing health system. It implies a different arrangement of authority. However, its transformative effects cannot be assumed in advance. They are likely to depend on institutional capacity, sustainable financing, political continuity, territorial access, and coordination mechanisms that protect Indigenous authority rather than translating it into existing administrative routines. These conditions have been highlighted in comparative experiences and in analyses of intercultural governance, suggesting that implementation outcomes will hinge on how such structures are developed and maintained. A more balanced implementation agenda must therefore recognize institutional, financial, political, and territorial barriers as central conditions shaping feasibility, rather than treating them as secondary obstacles. Analyses of intercultural governance consistently show that these structural factors influence whether implementation efforts can move beyond procedural inclusion and support durable forms of Indigenous authority. The challenge is not simply to integrate SISPI into mental health services, but to allow SISPI to reveal and transform the limits of those services. If implementation is reduced to coordination protocols, service procedures, reporting formats, and technical compliance, SISPI may expand access while leaving the epistemic hierarchy intact.

 The financial dimension is crucial. The original article explicitly recognizes the absence of costing as a limitation for making implementation more feasible.^1^ This limitation should not be treated as secondary. Without budget lines, payment mechanisms, contracting models, workforce recognition, transport resources, interpretation, community monitoring, and protection of ancestral knowledge, intercultural policy remains declarative. A potential proof of concept could be the establishment of a protected intercultural mental health budget line co-defined with Indigenous authorities. Rather than funding only clinical encounters, such a line would need to consider resources for traditional authorities’ participation, territorial accompaniment, interpretation, community monitoring, harmonization practices, ethical safeguards for ancestral knowledge, and coordination time. Sustainable implementation requires financing criteria defined with Indigenous authorities, not only for clinical services but for traditional actions, community accompaniment, territorial practices, training, governance spaces, and evaluation. Similarly, contracting mechanisms would benefit from avoiding arrangements that position traditional care as a low-cost substitute for biomedical care or as a reimbursable equivalent of Western services. Otherwise, the system may recognize spiritual harmony symbolically while funding only biomedical responses.

 The same caution applies to mutual aid and community strategies. Recent work on mutual aid groups warns that formal integration into health systems can strengthen community care, but can also neutralize its transformative potential if these groups become mere complements to psychiatric services rather than spaces of agency, recognition, and epistemic dignity.^13^ The lesson for intercultural mental health is direct: incorporation is not the same as transformation. Ancestral knowledge, community support, and spiritual care should not be absorbed as low-cost extensions of underfunded systems. They must retain authority over their meanings, purposes, safeguards, and modes of evaluation.

 This has implications for training and research. Cultural humility is necessary because it frames intercultural work as a lifelong process of self-critique and non-paternalistic partnership rather than mastery over another culture.^14^ Structural competency is also necessary because it shifts attention from individual cultural difference to the institutional, legal, economic, territorial, and political structures that shape suffering and care.^15^ But neither cultural humility nor structural competency is sufficient if Indigenous experts remain peripheral. Intercultural implementation requires Indigenous authorities, traditional knowers, intercultural psychologists, Indigenous researchers, interpreters, and community representatives to participate as decision-makers. Their decision-making role could encompass areas such as problem definition, referral criteria, ethical safeguards, curricula, information systems, implementation outcomes, evaluation indicators, and research priorities.

 The implication for standardized global tools is not rejection but subordination to context. Public mental health programs have helped expand access to mental healthcare in non-specialist settings, but standardized tools cannot define the whole field of recovery in Indigenous territories. An intercultural indicator of recovery, for example, is unlikely to be captured solely through symptom reduction, service uptake, or referral completion. It could also incorporate community-defined evidence of restored harmony, family and collective reconnection, territorial continuity, and recognition of traditional authority within the care pathway. Such indicators could be co-developed through local governance structures and incorporated alongside conventional monitoring frameworks rather than replacing them. In this sense, the Riohacha route offers more than a local case. It shows that implementation becomes intercultural only when communities do not merely adapt to health systems, but health systems become accountable to community epistemologies.

 The central lesson is clear: Intercultural implementation is possible, but not by adding culture to unchanged systems. The article by Agudelo-Hernández and Giraldo-Álvarez shows the potential of coproduction to create politically relevant routes for mental health recovery.^1^ The next step is harder. Colombia may need to ensure that SISPI and similar routes are not treated as already consolidated achievements; their legal recognition is better understood as an opening rather than an endpoint. Colombia must also prevent them from becoming procedural interculturality: culturally marked, administratively organized, and epistemically subordinated. Genuine transformation requires Indigenous governance, protected knowledge, sustainable financing, and evaluation criteria capable of recognizing harmony, territorial relation, collective agency, and self-government as legitimate outcomes of mental health policy.

## Disclosure of artificial intelligence (AI) use

 The authors used generative AI tools [ChatGPT] exclusively for language editing and manuscript preparation. All scientific content, analysis, interpretation, and conclusions remain the sole responsibility of the authors.

## Ethical issues

 Not applicable.

## Conflicts of interest

 Authors declare that they have no conflicts of interest.
